# Socioeconomic inequalities in Health: Reflections on the academic production from Brazil

**DOI:** 10.1186/s12939-016-0456-z

**Published:** 2016-11-17

**Authors:** Cesar Victora

**Affiliations:** International Center for Equity in Health, Federal University of Pelotas, Pelotas, Brazil

Since colonial times, Brazilian society has been characterized by a perverse concentration of wealth in the hands of a small elite. Ethnic group inequities were superimposed upon the concentration of wealth, with the near extermination of the native population and the massive slave trade. On top of socioeconomic and ethnic disparities, gender inequities also play an important role in Brazilian society.

Since international rankings of income concentration became available (http://www.tradingeconomics.com/brazil/gini-index-wb-data.html), Brazil has been consistently placed among the 10 most unequal countries in the world, and has often occupied the first position. Our Gini index showed significant declines in the past two decades, and such a reduction in inequality, jointly with the implementation of the Sistema Único de Saúde (National Health System), is deemed to have been one of the major drivers of the improved health status of the Brazilian population [1]. The massive economic inequality has had an impact on our academic production. For anyone working in the area of Public Health in Brazil, the role of social determinants, and in particular the magnitude of the health gap among the rich and the poor, are too evident to be ignored. As a result, Brazilian academics have produced a rich literature on the area of social determinants and health inequalities [2, 3].

In this commentary, I provide a brief personal view of the evolution of the study of health inequalities in our country, and discuss how the contents of the present journal supplement reflect our academic production.

Up to 1980, the quantification of socioeconomic position at individual or household level was uncommon. I recall two landmark articles from the 1970’s: an ecological study by Jairnilson Paim correlating indicators of poverty to health outcomes with States as the units of analysis, [4] and Carlos Monteiro’s survey on child nutrition according to land ownership in rural São Paulo State [5]. Both of these articles inspired my PhD thesis, which used multiple linear regression—a state-of-the-art approach at that time—to relate child health and nutrition to land tenure patterns in my home state of Rio Grande do Sul [6].

At that time, the dominance of academics with social sciences background in Public Health led to a great degree of mistrust regarding the use of quantitative methods for assessing inequalities. I remember that when presenting my PhD thesis at a national conference in the 1980’s, I was accused of being a “positivist”. Over time, quantitative studies gained wider acceptance, but were still strongly influenced by Marxist literature, as for example by attempting to adapt the concept of social class in our analyses [7–9]. These classifications resulted in several categories (e.g. underproletariat, typical proletariat, small bourgeoisie, etc.) and required combining information on occupation, qualifications, employment status and other variables. Because most of the information was based on open questions, classification of individuals had to be done manually and the process was very time consuming. Over time, these classifications were abandoned, even though several analyses showed that they were strongly associated with health outcomes.

Over time, more and more Brazilian studies relied on classifications of “social class” adopted in market research, particularly the one proposed by the Associação Brasileira de Empresas de Pequisa or ABEP (http://www.abep.org/criterio-brasil). Earlier versions of the scale classified families into classes from A to E, whereas the present scale has seven strata, from E1 to E7, [10]. The scale is based on an additive index that combines the number of several household assets (television, car, freezer, etc… including also the presence of full-time housekeepers), educational level of the head of household and characteristics of the neighborhood (paved streets, piped water). Each component of the index is assigned an a-priori, arbitrary weight; for example, having a university degree receives the same weight as having two housekeepers, or having two bathrooms in the house. Not surprisingly, such indices have been criticized due to their lack of a theoretical basis, yet they have been used frequently in analyses of health status and utilization of services, and have consistently revealed important inequalities. A good example is the article by Boccolini and Souza Jr in the present supplement [11].

In the global health literature, particular in maternal and child health, wealth indices derived through principal component analyses of household assets and characteristics of the house became immensely popular [12, 13]. The first component resulting from such analyses is then broken down into equal sized categories, usually quintiles. Unlike ABEP, these indices do not include components such as education of the head of the family or number of servants, being solely based on observable items, and in addition do no depend on arbitrary weights. If several assets that reflect wealth in a particular population are included, the indices are robust, being able to classify households with satisfactory accuracy. Over time, assets associated with wealth will change—for example, radios are hardly associated with wealth in most modern countries—but the classification into quintiles is hardly affected if relevant assets are included as the index is updated. The index reflects accumulated wealth, rather than recent earnings, and has the additional advantage or resulting in equal sized groups thus increasing statistical power and allowing comparisons over time and across populations. The article by França et al. in the present supplement relies on these indices to compare time trends in reproductive and maternal health from 1986 to 2013 [14]. And two other articles rely on such indices to document inequalities in care for the elderly [15] and in adult health care [16].

Among all articles included in this supplement, schooling was the most frequently used stratifier, being adopted in ten articles. Schooling is relatively easy to measure and requires a couple of questions. In international surveys this variable is often reported at the number of years attended, but in Brazil grade retention is common and it is usually measure as the highest grade successfully completed. When analyzing schooling data, there are additional issues including the large number of ties at specific values (such as 11 years which corresponds to full secondary education) and the fact that some subjects are still attending school (leading to censored data). Also, when studying time trends, it is essential to note that the size of a given category (for example, lack of any schooling) may change over time. For example, if 10% of subjects in the first point in time, and 5% in the second point belonged to this category, it is likely that the latter 5% will represent a more extreme group of the population than the original 10%, possibly with worse health status. Use of summary measures such as the slope index of inequality [17] can solve this problem by taking into account the whole distribution of the sample in terms of schooling, rather than relying on the comparison of extreme groups. Summing up, schooling is a useful stratification variable, but I currently prefer wealth stratification based on asset indices which allow us to work with equal-sized groups, particularly in global health where in some countries a large proportion of individuals fall in the “no schooling” or “incomplete primary” categories.

Household income is frequently used in Brazilian censuses and surveys, and represents the main type of socioeconomic stratification routinely adopted by the National Institute of Geography and Statistics (www.ibge.gov﻿.br). Common sense states that during household interviews the rich tend to underreport their actual income, while the poor do the opposite. In addition, income information is usually collected for the month before the survey, and therefore may show important variability over time for some employment categories, not to speak of the difficulty in measuring income in rural areas where self-produced foods account for a large proportion of the diet. In spite of these limitations, Brazilian society shows such wide income inequalities that studies relying on self-report income consistently show important health gaps between rich and poor. None of the studies included in the supplement used income as a stratifier, because this variable was not yet available in the survey database to which the authors had access to.

In addition to the socioeconomic stratifiers listed above, most articles also include breakdowns by sex and ethnic group, both of which are important dimensions of inequality that are superimposed upon, and may interact with, socioeconomic differences. In the Brazilian literature, self-reported skin color is used as a proxy for ethnicity, and recent research using genetic markers confirm that self-report is strongly correlated to African and European ancestry [18].

To conclude, I would like to thank the International Journal for Equity in Health, and particularly the guest editors for this supplement, for this important contribution to the literature on health inequalities. At this very moment, Brazil is undergoing profound political changes that will certainly impact on health, education and other social programs. Continued monitoring of inequalities in health is now more important than ever before, in order to document trends and provide reliable data to be fed back to politicians and—more importantly—to civil society.
